# Meperidine Induced Seizure in a Patient With Lyme Borreliosis

**DOI:** 10.4021/jocmr2009.12.1277

**Published:** 2009-12-28

**Authors:** Hatice Evren Eker, Hatice Izmirli, Sule Akin, Nesrin Bozdogan Ozyilkan, Anis Aribogan, Gulnaz Arslan

**Affiliations:** a Baskent University, Faculty of Medicine, Department of Anaesthesiology, Adana, Turkey; b Baskent University, Faculty of Medicine, Department of Anaesthesiology, Ankara, Turkey

## Abstract

A 15 years old child with Lyme borreliosis was treated with meperidine via a patient controlled analgesia (PCA) pump for pain management. He had no history of seizure and had normal hepatic and renal functions. At the 7th hour of meperidine PCA delivery, generalized tonic-clonic seizure was developed and successfully suppressed with antiepileptics and no neurologic sequel was occurred. The total meperidine consumption in the patient was quite lower than the recommended doses with PCA. Although Lyme disease might also cause seizure activities, the timing of the seizures was related with the accumulation of normeperidine which is the main metabolite of meperidine with central nervous system stimulant effect. The meperidine pain management on patients with Lyme syndrome should be reconsidered to avoid undesired effects.

Keywords

## Introduction

Lyme borreliosis is a tick-borne spirochetal infection with Borrelia burgdorferi which affects the skin, joints, heart and nervous system. Acute transverse myelitis, meningoradiculitis with pain, paraesthesia, and cranial nerve palsies and seizures with regional leptomeningeal enhancement are the leading symptoms of children with neuroborreliosis [1]. Epileptic seizures are poorly understood manifestations of this demyelinating disease and cerebral vasculitis that are disclosed on MRI (Magnetic resonance imaging) as ischemic foci which are substantiated for seizures in Lyme disease [2].

The neurological symptoms develop in 10 - 20% of children with borreliosis, and epileptic seizures in these children have only been described sporadically [3]. But it is well-known that the predisposition to seizure activity increases in patients with borreliosis when accompanied symptoms of central nervous system dysfunction occur. Besides, medical therapies with different epileptogenic potentials like meperidine, sevoflurane, clozapine, phenotiazines and cyclosporine can increase central nervous system excitability by their activated metabolites or contribute to reducing the seizure threshold [4]. Therefore, the routine use of these drugs in patients under the risk of epileptic seizures should be reconsidered to prevent the complicity of differential diagnosis during the essentially expected seizure attacks.

In this report, we described the case of generalized tonic-clonic seizures induced by patient controlled analgesia (PCA) with meperidine for pain management due to tonic spasms secondary to demyelination in a 15 year-old patient with Lyme syndrome who had no seizures during the initial examinations.

## Case Report

A 15 years old patient (BW: 55 kg) was diagnosed as Lyme disease according to serological studies for B. burgdorferi (ELISA, Western blot) which were positive for IgG in serum and CSF. His initial symptoms were aching pain, especially in the legs with proximal weakness of the lower extremities. He had no previous history of seizure and had normal hepatic and renal functions. The patient was hospitalized for antibiotherapy and control of diffuse muscle pains and treated with ceftriaxon and paracetamol. The patient was referred to the pain management service for appropriate medical treatment as his NRS (numeric rating scale) score was still 9.

The medical pain therapy was ordered with meperidine PCA and the pump was set to inject 5 mg/hour basal infusion, 5 mg bolus with a lockout time of 30 minutes. Following the first 30 minutes with initial bolus injection, the NRS score was decreased to 3 and at the 7th hour of the administration he was found in his bed having a generalized tonic-clonic seizure and the attack was successfully suppressed by 0.1 mg/kg midazolam and 10 mg/kg phenytoin. Since remaining meperidine, the patient had no further seizures, but because of the suspicion that Lyme syndrome could be responsible for triggering seizures, the pain therapy with meperidine was continued.

The epileptic attack was repeated 3 hours after the first generalized tonic-clonic seizure and was again successfully suppressed by phenytoin monotherapy, and meperidine infusion was terminated temporarily. Since remaining off meperidine, the patient had experienced no further seizures and regained consciousness without focal neurologic findings, and meperidine therapy was terminated definitely. The pain therapy was re-arranged with paracetamol (500 mg, infusion) and 15 drops of tramadol daily.

## Discussion

Lyme borreliosis is a multisystem disease associated with cranial nerve palsies, peripheral and cranial radiculopaties, aseptic menengitidis, encephalatic symptoms, seizures and demyelinating polyneuropathy. Vasculitis is thought to be the primary pathophysiological mechanism for focal cerebral lesions and its clinical reflection occurs as seizures. There is also raising suspicion that borreliosis itself could be responsible for triggering seizures [1].

The clinical manifestations of Lyme borreliosis differ from patient to patient, and most symptoms occur rarely. Painful meningoradiculitis and paraesthesia is one of the rarely occurred symptoms. The treatment modalities for pain especially in pediatric patients sustain special practice, and the main target in pain management has to contain both successful medical choices and any adverse effects.

Meperidine, through the medical choices, is one of the most commonly prescribed opioid analgesic with its high central nervous system depressant effects. Unlike this property, its main metabolite, normeperidine, is a central nervous system excitatory agent. Despite the fact that normeperidine has half the analgesic potency, it has twice neurotoxic effect of meperidine, and its accumulation can lead to symptoms of central nervous system toxicity such as nervousness, hyperreflexia, tremors, myoclonus, and generalized seizures with repeated use. These effects are nonopioid in nature and they can not be reversed by opioid antagonist administration [5].

The identified risk factors for the development of meperidine-related seizures include renal failure, high meperidine dosages, and co-administration of hepatic enzyme inducing medications or phenothiazines. However, healthy patients with normal renal function might also manifest to seizure activity due to rapid accumulation of normeperidine or might contribute by reducing the seizure threshold in specified disease with demyalinization [6,7].

Through the previous reports, meperidine related seizures associated with PCA pumps in healthy adolescents and young patients, with normal renal function and without the history of epilepsy, have been reported. In one of these healthy adolescents, a total of 1110 mg meperidine was received for postoperative pain control during the first 23 hours. Thirty minute after discontinuation of the PCA, the patient was noted to exhibit tonic-clonic movements. The serum meperidine concentration was measured 1418 ng.ml^-1^ while the therapeutic serum meperidine ranges were between 200 and 800 ng.ml^-1^. One month later, the patient received again meperidine via PCA, but this time the patient received only a total of 491 mg meperidine without any sign of central nervous system excitation [8]. This patient experienced a seizure after standard therapeutic doses of meperidine PCA and had no previously identified risk factors for the development of meperidine induced seizures. Our patient received a total of 110 mg meperidine within 7 hours which was approximately the tenth amount of the previously represented adolescents dose, and that was also quite lower than the recommended doses with PCA but it was sufficient for generalized tonic-clonic seizure occurrence.

Seizures in an Alzheimer's disease patient as a complication of colonoscopy premedication with meperidine have also been reported and attributed to the neuronal changes which occur during the progress of the illness as it was experienced in our patient [7]. The neural changes which occur in Lyme syndrome can themselves also predispose to seizures in this kind of rarely experienced cases. Although the underlying pathology and the changes due to the disease itself might also be supposed to the causes of seizures activity in our patient, the timing of the seizures was suspended from this potential possibility and we thought that the main cause of the seizures was probably due to the accumulation of normeperidine and its central nervous system stimulant effect.

In conclusion, this is the first case report of meperidine acting as the precipitating factor for generalized tonic-clonic seizures in a child with Lyme disease. We recommend avoidance of meperidine for pain management on patients with Lyme syndrome and on any patient with a condition that predisposes to seizure, to not to complicate the differential diagnosis and we suggest the use of alternative opioids and adjuvants for the pain management.
